# Calcium acamprosate: a triclinic polymorph

**DOI:** 10.1107/S1600536811046940

**Published:** 2011-11-09

**Authors:** Elisabetta Maccaroni, Walter Panzeri, Luciana Malpezzi

**Affiliations:** a Department of Chemistry, Materials and Chemical Engineering "G. Natta", Politecnico di Milano, Via Mancinelli 7, I-20131 Milano, Italy; b Istituto di Chimica del Riconoscimento Molecolare sezione "A. Quilico", CNR, Via Mancinelli 7, I-20131 Milano, Italy

## Abstract

The title compound, poly[bis­(μ_3_-4-acetamido­propane­sulfon­ato)­calcium], [Ca(C_5_H_10_NO_4_S)_2_]_*n*_, is a triclinic polymorph of the previously reported monoclinic structure [Toffoli *et al.* (1988 ▶). *Acta Cryst.* C**44**, 1493–1494]. The triclinic modification was found to have an all-*trans* configuration of the acetamido­propane chain, in contrast with the monoclinic polymorph which shows an angle of 74.66 (8)° between the S—C—C—C chain plane and that of the amide group. The Ca^2+^ cation is situated on an inversion centre and is hexa­coordinated by six O atoms belonging to different anions in a distorted octa­hedral geometry. This arrangement leads to a layered structure parallel to (011). The layers are held together by N—H⋯O hydrogen bonds and by short C—H⋯O inter­actions, both involving the sulfonate O atoms not coordinated to the Ca^2+^ cations. The structure was determined from a crystal twinned by non-merohedry [twin law (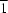
00, 0
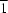
0, −0.335 −0.85 1), with a fractional contribution of the minor twin domain of 46.7 (1)%].

## Related literature

For the characterization of the monclinic polymorph and related structures, see: Toffoli *et al.* (1988 ▶). The title compound is a drug used successfully in the treatment of alcoholism. For the synthesis, see: Laboratorio Chimico Inter­nazionale SpA (2010 ▶). For its therapeutic effect and a tolerability study, see: Rösner *et al.* (2010 ▶). For proposed mechanisms of action, see: De Witte *et al.* (2005 ▶). Programs used for identifying the twin system were *PLATON* (Spek, 2009 ▶) and *CELL_NOW* (Bruker, 2008 ▶). For standard bond lengths, see: Allen *et al.* (1987 ▶).
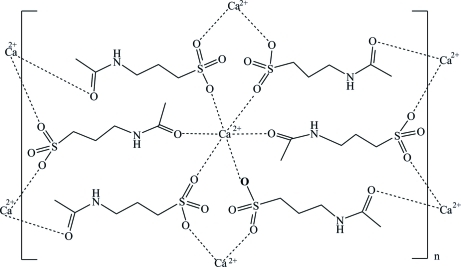


## Experimental

### 

#### Crystal data

[Ca(C_5_H_10_NO_4_S)_2_]
*M* *_r_* = 400.48
Triclinic, 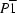

*a* = 5.5372 (4) Å
*b* = 8.1487 (6) Å
*c* = 9.7578 (7) Å
α = 69.159 (1)°
β = 84.305 (2)°
γ = 89.329 (2)°
*V* = 409.31 (5) Å^3^
*Z* = 1
Mo *K*α radiation
μ = 0.68 mm^−1^
*T* = 293 K
0.29 × 0.23 × 0.08 mm

#### Data collection

Bruker SMART APEX CCD diffractometer
Absorption correction: multi-scan (*TWINABS*; Bruker, 2008 ▶) *T*_min_ = 0.827, *T*_max_ = 0.948
22542 measured reflections
8721 independent reflections
7647 reflections with *I* > 2σ(*I*)
*R*_int_ = 0.027

#### Refinement

*R*[*F* ^2^ > 2σ(*F* ^2^)] = 0.034
*wR*(*F* ^2^) = 0.088
*S* = 1.02
8721 reflections
109 parameters
H-atom parameters constrained
Δρ_max_ = 0.39 e Å^−3^
Δρ_min_ = −0.33 e Å^−3^

### 

Data collection: *SMART* (Bruker, 2003 ▶); cell refinement: *SAINT* (Bruker, 2003 ▶); data reduction: *SAINT*; program(s) used to solve structure: *SIR97* (Altomare *et al.*, 1999 ▶); program(s) used to refine structure: *SHELXL97* (Sheldrick, 2008 ▶); molecular graphics: *SHELXTL/NT* (Sheldrick, 2008 ▶); software used to prepare material for publication: *SHELXL97*.

## Supplementary Material

Crystal structure: contains datablock(s) global, I. DOI: 10.1107/S1600536811046940/zl2412sup1.cif

Structure factors: contains datablock(s) I. DOI: 10.1107/S1600536811046940/zl2412Isup2.hkl

Additional supplementary materials:  crystallographic information; 3D view; checkCIF report

## Figures and Tables

**Table 1:** Hydrogen-bond geometry (Å, °)

*D*—H⋯*A*	*D*—H	H⋯*A*	*D*⋯*A*	*D*—H⋯*A*
N—H1*N*⋯O1^i^	0.86	2.15	3.0025 (12)	169
C5—H5*C*⋯O1^i^	0.96	2.48	3.3569 (15)	152
C1—H1*B*⋯O1^ii^	0.97	2.53	3.3007 (14)	137

Symmetry codes: (i) 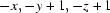; (ii) 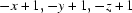.

**Fig. 1.**
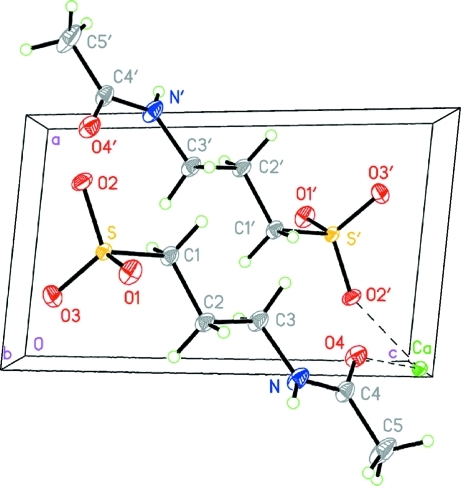
A view of the calcium acamprosate salt (B), showing the anion and cation of the asymmetric unit and the second anion, labeled with a prime symbol, generated by the symmetry operation [1 - x, 2 - y, 1 - z], to complete the unit formula.

**Fig. 2.**
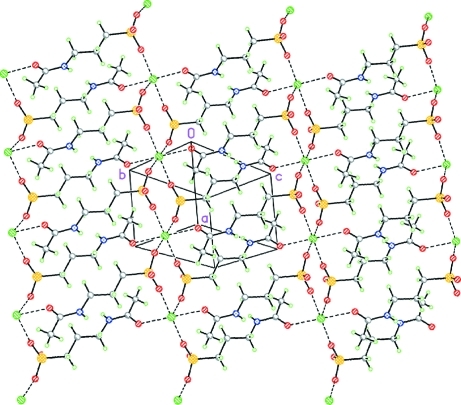
A view of the crystal packing of calcium acamprosate (B), showing a layer of the two dimensional structure running parallel to the (011) plane.

**Fig. 3.**
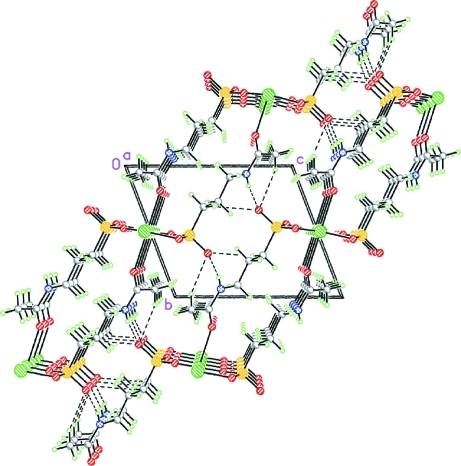
Crystal packing of calcium acamprosate (B), viewed along a axis. N—H···O1 hydrogen bonds and C—H···O1 short interactions are shown as dashed lines.

## supplementary crystallographic information

### Comment

Calcium acamprosate (Fig. 1), systematic name: calcium bis(3-acetylamino-propane)-1-sulphonate, also known as *N*-acetyl homotaurine, is a white crystalline synthetic compound which crystallizes as discrete acetylamino-propane-sulphonate anions, (C_5_H_10_N O_4_S)^-^, and Ca^2+^ cations, connected by Ca···O interactions. It is used in the treatment of alcoholism and it is specifically indicated for the maintenance of abstinence from alcohol in patients with alcohol dependence (Rösner *et al.*, 2010). The mechanism of action of calcium acamprosate in prevention of relapses is not completely understood, but it is believed to restore the normal chemical balance between neuronal excitation and inhibition that would be disrupted by long-term or chronic alcohol abuse. In other words, it helps the brain begin working normally again (De Witte *et al.*, 2005).

A monoclinic polymorph (A) of the title compound has been previously reported (Toffoli *et al.* 1988). The new polymorph form (B) crystallizes in the centrosymmetric *P*1 space group with the Ca^2+^ located on a crystallographic inversion centre, so there is only one single anion in the asymmetric unit of the elementary cell. The conformation of the acetylamino-propane chain shows the main geometric difference between polymorph A and polymorph B: in the triclinic modification (B) the conformation is all *trans*, while in the monoclinic form (A) an angle of 74.66 (8)° between the S—C—C—C chain plane and that of the amide group was found. Bond lengths and angles are within normal ranges (Allen *et al.*, 1987) and are comparable with those of polymorph (A). Each Ca^2+^ cation is coordinated in a distorted octahedral geometry to six different anions *via* Ca···O interactions with four sulfonyl O atoms and two carbonyl O atoms with a Ca···O distance in the range 2.283 (1)–2.394 (1) Å. The coordination of the Ca^2+^ cations to two terminal sides of the anions leads to a polymeric bidimensional structure extended in layers parallel to the (011) plane. The layers are connected into a three-dimensional network by N—H···O hydrogen bonds and by short C—H···O interactions, both involving the sulfonyl O atoms not coordinated to the Ca^2+^ cations.

### Experimental

The title compound was prepared according to the procedure patented by Laboratorio Chimico Internazionale SpA (2010). Polymorph B precipitates by adding 500 ml of isopropyl alcohol to a water solution of calcium acamprosate kept at 348–353 K and stirred for 53 h. The white solid obtained was filtered hot (348–353 K) and purified by washing with 300 ml of a 75/25 solution (preheated to 343 K) of isopropyl alcohol and deionized water. The wet product was dried under reduced pressure for 6 h at 318 K. Some crystals were suitable for single-crystal XR analysis. The XRPD pattern, carried out on the crystalline powder of this product, is completely in agreement with the powder pattern calculated from the data obtained by single-crystal XR analysis, thus confirming the purity of the crystalline phase of the batch.

### Refinement

The crystal under investigation was found to be non-merohedrally twinned. All reflections for both domains (10284 total) were integrated using Saint, obtaining a number of 4143 reflections (1692 unique ones) for component 1 only (mean I/σ = 12.3), 4146 reflections (1702 unique ones) for component 2 only (mean I/σ = 12.1) and 1974 reflections (929 unique ones) involving both components (mean I/σ = 16.7). The twin law (-1 0 0 0 - 1 0 - 0.335 - 0.85 1) was obtained by the TwinRotMat routine of the *PLATON* software (Spek, 2009). The orientation matrices for the two components were identified using the program *CELL_NOW* (Bruker, 2008), with the two twin components resulting related by a 180° rotation around the reciprocal *c* axis. The reflection data were corrected for absorption using *TWINABS* (Bruker, 2008), obtaining the HKLF-5 type list of reflections (Sheldrick, 2008) with twin-contributor indicators. The structure was solved using direct methods with only the non-overlapping reflections of component 1 and refined with all reflections of component 1, including the overlapping ones. The fractional contribution of the minor twin component refined to 46.7 (1). H atoms were placed in calculated positions and refined in a riding model with C—H distances of 0.96–0.97 Å and and N—H distance of 0.86 Å. All H atoms were refined with *U*_iso_(H) values equal to 1.5 *U*_eq_ of the carrier atom for the methyl group and 1.2 *U*_eq_ for all remaining atoms.

### Crystal data

**Table:**

[Ca(C_5_H_10_NO_4_S)_2_]	*Z* = 1
*M**_r_* = 400.48	*F*(000) = 210
Triclinic, *P*1	*D*_x_ = 1.625 Mg m^−^^3^
Hall symbol: -P 1	Mo *K*α radiation, λ = 0.71069 Å
*a* = 5.5372 (4) Å	Cell parameters from 4793 reflections
*b* = 8.1487 (6) Å	θ = 2.3–27.1°
*c* = 9.7578 (7) Å	µ = 0.68 mm^−^^1^
α = 69.159 (1)°	*T* = 293 K
β = 84.305 (2)°	Plate, colourless
γ = 89.329 (2)°	0.29 × 0.23 × 0.08 mm
*V* = 409.31 (5) Å^3^	

### Data collection

**Table:**

Bruker SMART APEX CCD diffractometer	8721 independent reflections
Radiation source: fine-focus sealed tube	7647 reflections with *I* > 2σ(*I*)
graphite	*R*_int_ = 0.027
ω scans	θ_max_ = 27.5°, θ_min_ = 2.2°
Absorption correction: multi-scan (*TWINABS*; Bruker, 2008)	*h* = −7→7
*T*_min_ = 0.827, *T*_max_ = 0.948	*k* = −10→10
22542 measured reflections	*l* = −12→12

### Refinement

**Table:**

Refinement on *F*^2^	Primary atom site location: structure-invariant direct methods
Least-squares matrix: full	Secondary atom site location: difference Fourier map
*R*[*F*^2^ > 2σ(*F*^2^)] = 0.034	Hydrogen site location: inferred from neighbouring sites
*wR*(*F*^2^) = 0.088	H-atom parameters constrained
*S* = 1.02	*w* = 1/[σ^2^(*F*_o_^2^) + (0.0478*P*)^2^] where *P* = (*F*_o_^2^ + 2*F*_c_^2^)/3
8721 reflections	(Δ/σ)_max_ = 0.006
109 parameters	Δρ_max_ = 0.39 e Å^−^^3^
0 restraints	Δρ_min_ = −0.33 e Å^−^^3^

### Special details

**Table:**

Geometry. All e.s.d.'s (except the e.s.d. in the dihedral angle between two l.s. planes) are estimated using the full covariance matrix. The cell e.s.d.'s are taken into account individually in the estimation of e.s.d.'s in distances, angles and torsion angles; correlations between e.s.d.'s in cell parameters are only used when they are defined by crystal symmetry. An approximate (isotropic) treatment of cell e.s.d.'s is used for estimating e.s.d.'s involving l.s. planes.
Refinement. Refinement of *F*^2^ against ALL reflections. The weighted *R*-factor *wR* and goodness of fit *S* are based on *F*^2^, conventional *R*-factors *R* are based on *F*, with *F* set to zero for negative *F*^2^. The threshold expression of *F*^2^ > σ(*F*^2^) is used only for calculating *R*-factors(gt) *etc*. and is not relevant to the choice of reflections for refinement. *R*-factors based on *F*^2^ are statistically about twice as large as those based on *F*, and *R*- factors based on ALL data will be even larger.

### Fractional atomic coordinates and isotropic or equivalent isotropic displacement parameters (Å^2^)

**Table:**

	*x*	*y*	*z*	*U*_iso_*/*U*_eq_	
Ca	1.0000	0.5000	0.0000	0.02008 (8)	
S	0.46696 (4)	0.53055 (3)	0.23925 (3)	0.02053 (7)	
O1	0.39518 (14)	0.65813 (9)	0.30610 (8)	0.03333 (19)	
O2	0.71997 (12)	0.55429 (9)	0.17559 (8)	0.02862 (18)	
O3	0.30448 (15)	0.52304 (11)	0.13414 (8)	0.0383 (2)	
O4	0.06178 (14)	−0.20198 (9)	0.83520 (8)	0.0340 (2)	
N	−0.01015 (17)	0.07771 (11)	0.69942 (10)	0.0334 (2)	
H1N	−0.1087	0.1630	0.6866	0.040*	
C1	0.44992 (19)	0.32245 (13)	0.38187 (11)	0.0257 (2)	
H1A	0.4796	0.2327	0.3388	0.031*	
H1B	0.5769	0.3165	0.4450	0.031*	
C2	0.20568 (19)	0.28297 (13)	0.47557 (12)	0.0282 (2)	
H2A	0.1742	0.3719	0.5193	0.034*	
H2B	0.0776	0.2860	0.4138	0.034*	
C3	0.20577 (19)	0.10418 (14)	0.59580 (12)	0.0305 (3)	
H3A	0.2108	0.0138	0.5524	0.037*	
H3B	0.3493	0.0946	0.6474	0.037*	
C4	−0.0642 (2)	−0.07020 (14)	0.81257 (12)	0.0294 (3)	
C5	−0.2877 (2)	−0.07026 (17)	0.91136 (14)	0.0541 (4)	
H5A	−0.2469	−0.0998	1.0105	0.081*	
H5B	−0.4034	−0.1553	0.9080	0.081*	
H5C	−0.3566	0.0442	0.8791	0.081*	

### Atomic displacement parameters (Å^2^)

**Table:**

	*U*^11^	*U*^22^	*U*^33^	*U*^12^	*U*^13^	*U*^23^
Ca	0.01787 (15)	0.02063 (15)	0.01751 (15)	−0.00083 (11)	0.00031 (11)	−0.00214 (12)
S	0.01931 (13)	0.02153 (13)	0.01727 (13)	−0.00037 (10)	0.00084 (10)	−0.00333 (10)
O1	0.0406 (5)	0.0242 (4)	0.0332 (4)	0.0049 (3)	0.0042 (4)	−0.0100 (3)
O2	0.0207 (4)	0.0316 (4)	0.0274 (4)	−0.0034 (3)	0.0064 (3)	−0.0052 (3)
O3	0.0328 (4)	0.0520 (5)	0.0267 (4)	−0.0053 (4)	−0.0088 (4)	−0.0081 (4)
O4	0.0360 (5)	0.0221 (4)	0.0337 (5)	0.0035 (3)	0.0052 (4)	0.0001 (3)
N	0.0362 (6)	0.0223 (5)	0.0295 (5)	0.0062 (4)	0.0106 (4)	0.0018 (4)
C1	0.0271 (6)	0.0208 (5)	0.0229 (5)	0.0011 (4)	0.0026 (5)	−0.0016 (4)
C2	0.0254 (6)	0.0244 (6)	0.0265 (6)	−0.0001 (4)	0.0047 (5)	−0.0006 (5)
C3	0.0309 (6)	0.0235 (6)	0.0284 (6)	−0.0001 (4)	0.0065 (5)	−0.0011 (5)
C4	0.0339 (6)	0.0225 (6)	0.0247 (6)	−0.0003 (5)	0.0047 (5)	−0.0019 (5)
C5	0.0578 (9)	0.0355 (7)	0.0443 (8)	0.0108 (6)	0.0261 (7)	0.0076 (6)

### Geometric parameters (Å, °)

**Table:**

Ca—O3^i^	2.2828 (8)	N—C3	1.4523 (13)
Ca—O3^ii^	2.2828 (8)	N—H1N	0.8600
Ca—O2^iii^	2.3519 (7)	C1—C2	1.5245 (13)
Ca—O2	2.3519 (7)	C1—H1A	0.9700
Ca—O4^iv^	2.3941 (7)	C1—H1B	0.9700
Ca—O4^v^	2.3941 (7)	C2—C3	1.5109 (14)
S—O1	1.4442 (7)	C2—H2A	0.9700
S—O3	1.4471 (8)	C2—H2B	0.9700
S—O2	1.4609 (7)	C3—H3A	0.9700
S—C1	1.7662 (10)	C3—H3B	0.9700
O3—Ca^vi^	2.2828 (8)	C4—C5	1.4910 (16)
O4—C4	1.2382 (12)	C5—H5A	0.9600
O4—Ca^vii^	2.3941 (7)	C5—H5B	0.9600
N—C4	1.3256 (13)	C5—H5C	0.9600
			
O3^i^—Ca—O3^ii^	180.00 (4)	C2—C1—S	113.33 (7)
O3^i^—Ca—O2^iii^	88.69 (3)	C2—C1—H1A	108.9
O3^ii^—Ca—O2^iii^	91.31 (3)	S—C1—H1A	108.9
O3^i^—Ca—O2	91.31 (3)	C2—C1—H1B	108.9
O3^ii^—Ca—O2	88.69 (3)	S—C1—H1B	108.9
O2^iii^—Ca—O2	180.000 (1)	H1A—C1—H1B	107.7
O3^i^—Ca—O4^iv^	92.36 (3)	C3—C2—C1	110.28 (8)
O3^ii^—Ca—O4^iv^	87.64 (3)	C3—C2—H2A	109.6
O2^iii^—Ca—O4^iv^	97.35 (3)	C1—C2—H2A	109.6
O2—Ca—O4^iv^	82.65 (3)	C3—C2—H2B	109.6
O3^i^—Ca—O4^v^	87.64 (3)	C1—C2—H2B	109.6
O3^ii^—Ca—O4^v^	92.36 (3)	H2A—C2—H2B	108.1
O2^iii^—Ca—O4^v^	82.65 (3)	N—C3—C2	110.46 (8)
O2—Ca—O4^v^	97.35 (3)	N—C3—H3A	109.6
O4^iv^—Ca—O4^v^	180.00 (2)	C2—C3—H3A	109.6
O1—S—O3	112.42 (5)	N—C3—H3B	109.6
O1—S—O2	112.71 (5)	C2—C3—H3B	109.6
O3—S—O2	111.72 (5)	H3A—C3—H3B	108.1
O1—S—C1	107.12 (5)	O4—C4—N	122.27 (10)
O3—S—C1	106.99 (5)	O4—C4—C5	121.44 (10)
O2—S—C1	105.35 (5)	N—C4—C5	116.28 (9)
S—O2—Ca	144.39 (5)	C4—C5—H5A	109.5
S—O3—Ca^vi^	170.63 (5)	C4—C5—H5B	109.5
C4—O4—Ca^vii^	132.62 (7)	H5A—C5—H5B	109.5
C4—N—C3	123.86 (9)	C4—C5—H5C	109.5
C4—N—H1N	118.1	H5A—C5—H5C	109.5
C3—N—H1N	118.1	H5B—C5—H5C	109.5
			
O1—S—O2—Ca	−156.68 (7)	O2—S—C1—C2	172.54 (8)
O3—S—O2—Ca	−28.97 (9)	S—C1—C2—C3	−179.41 (8)
C1—S—O2—Ca	86.85 (8)	C4—N—C3—C2	176.87 (11)
O3^i^—Ca—O2—S	23.48 (8)	C1—C2—C3—N	170.74 (9)
O3^ii^—Ca—O2—S	−156.52 (8)	Ca^vii^—O4—C4—N	−169.56 (8)
O4^iv^—Ca—O2—S	−68.73 (8)	Ca^vii^—O4—C4—C5	9.90 (18)
O4^v^—Ca—O2—S	111.27 (8)	C3—N—C4—O4	−3.33 (19)
O1—S—C1—C2	52.32 (9)	C3—N—C4—C5	177.18 (11)
O3—S—C1—C2	−68.44 (9)		

Symmetry codes: (i) −*x*+1, −*y*+1, −*z*; (ii) *x*+1, *y*, *z*; (iii) −*x*+2, −*y*+1, −*z*; (iv) −*x*+1, −*y*, −*z*+1; (v) *x*+1, *y*+1, *z*−1; (vi) *x*−1, *y*, *z*; (vii) *x*−1, *y*−1, *z*+1.

### Hydrogen-bond geometry (Å, °)

**Table:**

*D*—H···*A*	*D*—H	H···*A*	*D*···*A*	*D*—H···*A*
N—H1N···O1^viii^	0.86	2.15	3.0025 (12)	169.
C5—H5C···O1^viii^	0.96	2.48	3.3569 (15)	152.
C1—H1B···O1^ix^	0.97	2.53	3.3007 (14)	137.

Symmetry codes: (viii) −*x*, −*y*+1, −*z*+1; (ix) −*x*+1, −*y*+1, −*z*+1.
